# Dominance relationships and coalitionary aggression against conspecifics in female carrion crows

**DOI:** 10.1038/s41598-019-52177-7

**Published:** 2019-11-04

**Authors:** Benedikt Holtmann, Julia Buskas, Matthew Steele, Kristaps Sokolovskis, Jochen B. W. Wolf

**Affiliations:** 1 Division of Evolutionary Biology, Department of Biology, LMU Munich, Großhaderner Straße 2, 82152 Planegg-Martinsried, Germany; 2 Behavioural Ecology Group, Department of Biology, LMU Munich, Großhaderner Straße 2, 82152 Planegg-Martinsried, Germany; 3 Department of Ecology and Genetics, Evolutionary Biology Centre, Uppsala University, Norbyvägen 14-18, 75236 Uppsala, Sweden

**Keywords:** Zoology, Animal behaviour

## Abstract

Cooperation is a prevailing feature of many animal systems. Coalitionary aggression, where a group of individuals engages in coordinated behaviour to the detriment of conspecific targets, is a form of cooperation involving complex social interactions. To date, evidence has been dominated by studies in humans and other primates with a clear bias towards studies of male-male coalitions. We here characterize coalitionary aggression behaviour in a group of female carrion crows consisting of recruitment, coordinated chase, and attack. The individual of highest social rank liaised with the second most dominant individual to engage in coordinated chase and attack of a lower ranked crow on several occasions. Despite active intervention by the third most highly ranked individual opposing the offenders, the attack finally resulted in the death of the victim. All individuals were unrelated, of the same sex, and naïve to the behaviour excluding kinship, reproduction, and social learning as possible drivers. Instead, the coalition may reflect a strategy of the dominant individual to secure long-term social benefits. Overall, the study provides evidence that members of the crow family engage in coordinated alliances directed against conspecifics as a possible means to manipulate their social environment.

## Introduction

Cooperation between individuals is commonplace in humans and widespread across the animal kingdom^1,2^. It ranges from cooperation between parents sharing the burden of brood care to complex interactions among several members of group living species^3–5^. Coalitionary aggression constitutes a prominent example for the latter: two or more individuals actively engage in cooperative behaviour inflicting damage or other costs upon a conspecific target to their own benefit^3–6^. For instance, adult male chimpanzees (*Pan troglodytes*) have been shown to form coalitions to attack other males with the possible benefit of increasing their social rank and access to mates^7,8^.

The successful formation of such coalitions requires strategic decisions as to the choice of the appropriate interaction partner and antagonist^9^. Coalitionary aggression is likely to occur in species disposing of the cognitive abilities necessary for individual recognition, insight into tertiary relationships, imagination and prospection. Not surprisingly, evidence of coalitionary aggression and other complex social behaviours is dominated by species with advanced social cognition such as humans and their closest primate relatives^10–13^. During the last decades, however, evidence for complex social behaviours has been accumulating in other animals^14^. For example, the ability to recognize other’s relationships, draw social inferences, and use this information to project behavioural decisions has been documented in hyenas, dolphins, and birds^15–17^. Cognitive abilities and social interactions within the crow family (Corvidae), in particular, shows striking analogy to primates^14,18^. Rooks (*Corvus frugilegus*) and ravens (*Corvus corax*), for instance, develop close, non-sexual relationships with other conspecifics^19–21^. Individuals spend disproportionality much time in close proximity to specific partners, share affiliative behaviours (e.g. preening), cooperate or provide agonistic support (i.e. individuals support a partner in an on-going conflict;^19–21^). Individual recognition, selective attention and insight into tertiary social relationships^22,23^ allows corvids, in principle, to engage in complex social interactions and manipulate their social environment^24^.

Carrion crows (*Corvus (corone) corone*) are members of the corvid genus *Corvus* comprising crows, ravens, rooks and jackdaws^25^. Sexually immature individuals form constantly changing flocks of which small groups or individuals disperse, socialize, and forage during the day, but gather at communal roosts at night^26^. The flexible social organization and the opportunity of repeated interaction with conspecifics in non-breeding flocks provides a social environment conducive to complex social interaction^26,27^. While carrion crows can on rare occasions reach physiological sexual maturity at an age of one year, they normally remain in non-breeding groups for 3–5 years before they occupy breeding territories and form lifelong pairs^26^.

Here, we document an incidence of coalitionary aggression within a single-sex female group of unrelated, sexually immature carrion crows. We characterize the social behavioural components leading to the cooperation in detail, and unravel its intimate association with social structure of the group and dominance status of the individuals.

## Results

### Coalitionary aggression

Behavioural interactions within a group of immature, female carrion crows were monitored with the help of video recordings. Repeated recordings at approximately monthly intervals allowed quantifying temporal dynamics of social relationships within the group. The five recordings contained three incidences of coalitionary aggression distributed over a 1.5-hour period. This behaviour was characterized post-hoc from the video footage. It was neither experimentally induced, nor foreshadowed by altered behavioural patterns visible to the staff who had closely monitored the group of crows for over a year. In brief, the two most dominant females, C45 (red) and C59 (yellow), repeatedly engaged in a coordinated attack against one of the lower ranked individuals C58 (green). On each occasion, the two aggressors synchronized their behaviour using a specific sequence of vocal and non-vocal communication. The behavioural sequence of the three recorded attacks can be partitioned into three components: 1) recruitment, 2) chase, and 3) the attack itself (Supplementary videos 1–3).

1. *Recruitment*. Shortly prior to the attacks (8–32 seconds), the dominant individual, red, initiated interaction with yellow by approach and repeated bouts of characteristic vocalization (Fig. 1a; Supplementary videos 1–2). Yellow reacted with two calls accompanied by bowing and tail fanning. Finally, red emitted five “oui”, calls that we had never encountered in any other context within the investigated five recordings nor during other behavioural tests or daily husbandry (Supplementary audio 1). These calls were accompanied by a threatening gesture of raised crown and mantle feathers and the beak pointed down^26^. The response by yellow initiated the second phase.

**Figure 1:**
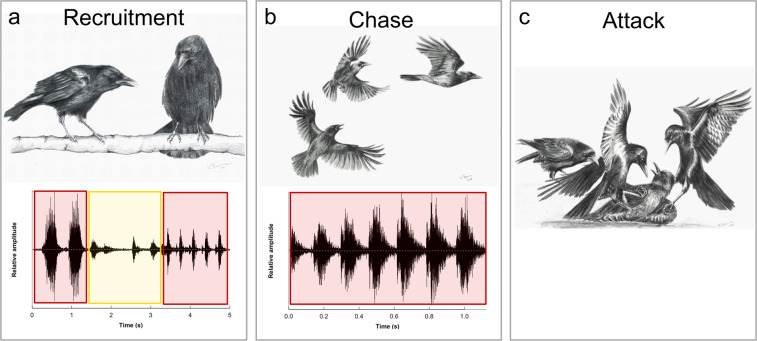
Sequence of behaviours and vocalizations characterizing coalitionary aggression within a social group of unrelated, sexually immature female carrion crows. (**a**) The dominant individual C45 (left) recruits the second most dominant individual C59 (right). The recruitment is characterized by social bonding behaviour and a series of specific vocalizations between C45 (red) and C59 (yellow). (**b**) Initiated by a vocal signal of C45 the allies engage in a coordinated chase of the subordinate victim (C58, upper right). (**c**) Both aggressors immobilize the victim while pecking ferociously at its head. A fourth bird (C29, left) intervenes, presumably to the assistance of the victim, by pulling on one of the attackers’ wings. Acoustic time waves in panel a and b were created using the *seawave* package^50^ in *R*^51^. Drawings courtesy of Kristina Fraune.
2. *Chase*. After recruitment, one of the two dominant individuals (red or yellow) positioned itself high up in the aviary (e.g. crossbar under the roof) facing the victim. On most occasions, yellow actively started chasing the victim, while red observed from a distance. In the third and most severe attack red initiated the chase by a sharp call. Immediately prior to joining the chase, red expressed a distinct rattle call (Fig. 1b) with the apparent effect of coordinating and synchronising the partner’s behaviour with her own^28^: the two aggressors pursued the victim from alternating positions at opposite ends of the aviary (Supplementary video 2). This ‘pincer tactic’ has been observed in wild breeding pairs of carrion crows when defending their territory against intruders^29^.
3. *Attack*. In occasions when one of the attackers managed to immobilize green on the ground the second aggressor joined immediately. During the first two assaults, green was able to free herself and escaped before the second aggressor could get hold. During the third attack, however, the victim was not able to escape and both attackers used their claws to immobilize the victim and their bills to ferociously peck at green’s head (Fig. 1c; Supplementary video 3). Shortly after, the individual C29 (blue), ranked third in the group, entered the fight and provided agonistic support to the victim (Supplementary video 3). Calling loudly for a few seconds to no avail, it repeatedly pecked one of the attackers (yellow). As yellow showed no reaction and continued its aggression against the victim, blue pulled on the other attacker’s (red) wings and pecked. Finally, blue flew up and down the aviary calling loudly with the effect that yellow stopped attacking the victim and approached blue. Blue raised and flapped her wings at yellow who in response jabbed its bill at blue, and then returned to join red in pecking veraciously at green’s head. Moments later, green escaped and the two attackers stopped following their victim, which was found dead the next morning. On top of that, a second individual C27 (grey) was found dead two days later. Pathological examination of the two corpses suggested traumatic injuries as the most likely cause of death of the otherwise healthy individuals (Supplementary Fig. S1).

### Dominance hierarchy and inter-individual relationships

**Figure 2:**
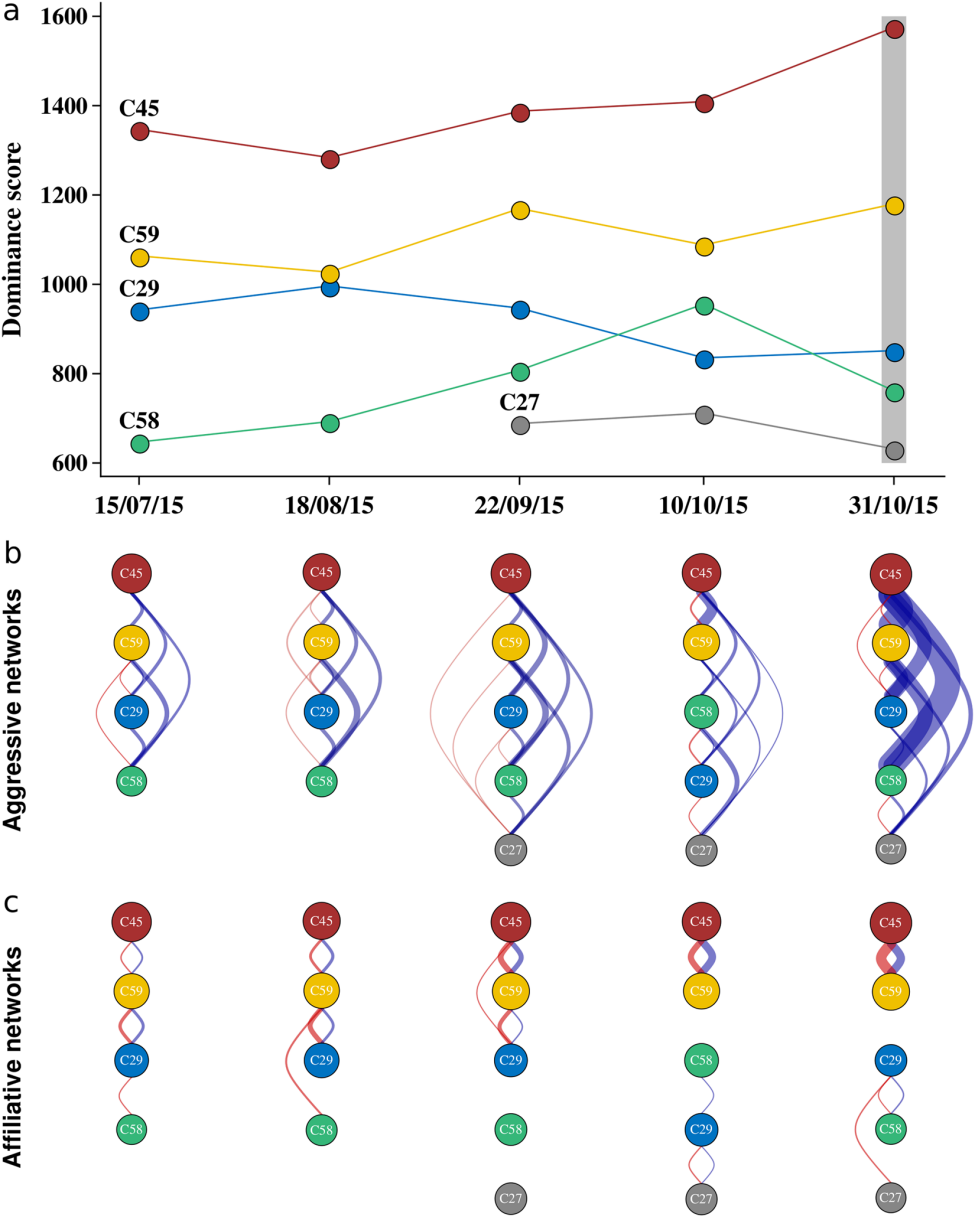
Dominance hierarchies and social networks in a group of female carrion crows through time. (**a**) Change of dominance hierarchies as inferred by Elo-scores. The vertical grey rectangle indicates incidences of coalitionary aggression, in which C45 (red) and C59 (yellow) cooperatively attacked individual C58 (green). (**b**,**c**) Social networks of (**b**) aggressive and (**c**) affiliative interactions for each observation. The order and size of the nodes (circles) is proportional to individual dominance ranks. Edges (lines between individuals) represent interactions, with edges to the right of networks (blue) denoting higher-ranked individuals interacted with lower-ranked individuals, while edges on the left side of networks (in red) denote lower-ranked individuals interacted with higher-ranked individuals. Edge width reflects the number of interactions. Dominance hierarchies were visualised using the *R*^51^ package *ggplot2*^56^, and social networks were created using the *R* package *igraph*^60^.

Next, we considered the social context of the group. In total, we recorded 659 (mean ± SD, 131.8 ± 111.7 per video) agonistic interactions from which dominance hierarchies were inferred. Throughout the entire period of investigation, individual C45 (red) was uncontested as the dominant individual followed by C59 (yellow), C29 (blue), C58 (green) and the most submissive individual C27 (grey, Fig. 2a). Grey had been isolated for reasons of health and re-joined the group on the 22^nd^ of September. Shortly after, blue and green temporarily switched their ranks (Fig. 2a).

Social networks of agonistic and affiliative interactions shed further light on individual relationships. Each individual was involved in agonistic interactions with at least one other individual for the entire observational period (Fig. 2b). Concomitant with the re-introduction of grey the number of agonistic interactions increased substantially; in particular between the two dominant individuals, red and yellow, that more generally displayed most of the aggressive interactions (Fig. 2b). Importantly, red directed the majority of its aggressive behaviour towards yellow. Moreover, quadratic assignment procedure (QAP) correlation tests between networks that included the same individuals revealed that individuals predominantly directed aggressive behaviours towards the same conspecifics (networks 1–2 QAP: *r* = 0.97, *N* = 4, *p* = 0.042; networks 3–4 QAP: *r* = 0.43, *N* = 5, *p* = 0.07; networks 4–5 QAP: *r* = 0.71, *N* = 5, *p* = 0.007; Supplementary Fig. S2).

Affiliative behaviour was overall less frequently observed (total: 223, mean ± SD, 44.6 ± 13.1 per video), and was not initiated nor experienced by every female. Affiliative interactions were exchanged near-exclusively between the three dominant individuals by order of rank: red with yellow, yellow with blue (Fig. 2c). The direction and intensity of affiliative interactions was also less stable than for agonistic behaviour (QAP: all r > 0.84all *p* > 0.075; Fig. S2). Over the course of study, red and yellow developed an exclusive relationship supplanting any affiliative interaction between yellow and blue (Fig. 2c). Specifically, red actively intervened in attempts by blue to interact with yellow by placing herself in between or by simply displacing blue on occasions it tried to seek yellow’s proximity. Such interventions effectively monopolized red’s affiliation with yellow.

## Discussion

This study discloses evidence for lethal coalitionary aggression among females in a system other than humans^30^ or non-human apes^8,31^. It demonstrates that members of the crow family also engage in alliances directed against conspecifics^18^.

All instances of coalitionary aggression involved the same offenders (red, yellow) and victim (green). On each occasion, the dominant individual initiated the attack by a series of vocalizations and non-vocal social bonding behaviour towards the second most dominant individual. These observations provide tentative empirical support for the notion that the coalitionary attack was prompted by an active recruitment rather than the outcome of a by-product of individually motivated concurrent attacks by two crows. Moreover, the recruitment phase resembles social bonding behaviours and vocalizations described in other corvids using similar displays to strengthen their partnership^19^. Display behaviours unrelated to sexual contexts have been suggested to function as signals to manipulate a partner’s behaviour^32^. Thus, we interpret this behaviour as solicited alliance initiated by red reinforcing social bonds.

The recruitment was followed by a coordinated, synchronized chase, which finally resulted in the immobilization of the target. Successful completion of these behavioural components requires an understanding of tasks of both offenders to synchronize and conduct the cooperative chase from alternating positions. All individuals were hand-raised and naïve to the behaviour, excluding social learning from experienced conspecifics^33,34^ or experience projection as template^18^. While this could indicate that the capacity to engage in coordinated, aggressive coalitions is an innate strategy to manipulate social dominance, such an assumption remains to be empirically tested.

Importantly, carrion crows reach physiological sexual maturity generally at an age of 2–3 years and have their first brood between 3–5 years of age^26^. Thus, competition for sexual partners seems unlikely as reason for the observed behaviour. Moreover, all individuals were unrelated excluding kinship as possible motivational force. Physical attacks were never observed outside the context of an alliance and did not arise as a spontaneous reaction to preceding conflict between the individuals. The motivational state, therefore, appears not to derive from stimuli immediately preceding the attacks. A motivation rather ought to be sought in the long-term social group dynamics. With the necessary reservation of over-interpretation, we hypothesize that coalitionary aggression may function as a means for social niche construction and stabilization^35^ to the avail of potential future fitness benefits^7,36,37^. Future research with controlled experimental design is now needed to gain full understanding of the relationships between dominance status, social associations among individuals, and reproductive success. In corvids, where securing a breeding territory crucially depends on the social status within local bachelor groups, the link between dominance rank and future reproductive success seems plausible^26,38^.

Red was uncontested in its dominance during the entire study period. Cooperating with the second most dominant individual, yellow, likely increased the chances of a favourable outcome of the attack^39,40^. Notably, the coalition was formed in the wake of social instability with the victim (green) rising in rank. Concomitantly, red reinforced its affiliative interactions with yellow and actively intervened in bonding attempts between blue and yellow securing an affiliative relationship with yellow to the exclusion of all other individuals. Selective social intervention has similarly been observed in ravens where it likely functions to preclude the formation of coalitions between individuals that may challenge the dominant’s rank^24^. Consistent with the motivation to secure dominance status, red not only reinforced affiliation with yellow, but also significantly increased the number of agonistic interactions. The relationship between the two offenders thus was not mutual, but dominated by red. Strong asymmetry in hierarchies provide the opportunity for dominant individuals to exploit subordinates to achieve their own individual goals^41,42^. In keas (*Nestor notabilis*), for example, dominant individuals aggressively forced subordinates to open an apparatus to monopolize a food reward^43^. This raises that possibility that red may have coerced its subordinate partner to participate in the attacks to secure its own status^44^. The threat of punishment to enforce cooperation might therefore explain the participation of yellow in a potentially costly fight without gaining any immediate benefits. An alternative motivation may lie in the gain of social benefits, such as future reciprocation of agonistic support^21^.

Notably, coalitionary aggression in crows is not limited to captivity. We have ourselves observed an incidence in a group of seven wild carrion crows in Munich, Germany. Analogous to the description above, one individual initiated an attack, which was immediately joined by three other individuals (personal observation J. B. W. Wolf). While the confined space in captivity as well as the artificial group composition (females only) might have generated stress, which in turn may have facilitated the lethal outcome, joined aggression directed against conspecifics has likewise been shown to result in the death of the victim under unrestrained conditions in the wild (Supplementary video 4).

This study provides a detailed account of lethal coalitionary aggression against conspecifics in the long-term social context of a group of immature female carrion crows. Placing the behavioural sequence consisting of a recruitment phase, chase and attack into the context of long-term social dominance hierarchies in the group suggests that carrion crows may use information about social structure to actively manipulate their own social environment^18,39,42^. The study thus adds to a literature dominated by male-male interaction in primates and provides further evidence for behavioural repertoires reflecting social intelligence in birds^18,45^.

## Methods

### Population sampling and animal husbandry

Five female carrion crow nestlings were sampled from different nests near Konstanz, Germany (47°45N′, 9°10′E) at an age of 14–28 days during May 2014. Sex was determined genetically following the methods described by Griffith *et al*.^46^. To avoid any confounding effects of relatedness, only a single individual was selected from each nest. Assessment of genetic relatedness between individuals based on 1152 single nucleotide polymorphism data genotyped as part of a population study on the Golden Gate platform (Illumina) corroborated that all individuals were unrelated females^47^. After transfer of nestlings to Sweden by airplane, all crows were hand-raised indoors at Tovetorp field station, Sweden (58°56′N, 17°8′E). For individual identification, we banded each bird with a unique combination of one metal and two colour rings (Supplementary Table S1). At the time birds could feed independently, all individuals were released into a roofed outdoor enclosure (6.5 × 4.8 × 3.5 m) and housed together. One individual (C27, grey), which was limited in its flight ability from early days onward due to brittle primary wing feathers, was removed from the group after six month and kept in a different aviary for better monitoring of health. Grey was reintroduced to the group on the 22^nd^ of September 2015 (Supplementary Table S2). The aviaries were equipped with perches, green fresh foliage, and a variety of ground substrate, such as tree trunks, bark and wood chips. A separate compartment within the aviary provided hiding opportunities. Food (a mix of beef, eggs, fresh fruit and vegetables, peanuts, dry cat or dog food) was provided daily and was accessible *ad libitum*. For behavioural enrichment, we presented the food items in a varying set of puzzles (e.g. balls, ropes, cardboard boxes). Water for drinking and bathing was freely available.

### Behavioural observations

We videotaped the crows’ behaviours outside of the reproductive period randomly on eleven days between July and October 2015. From these eleven videos, we choose five that were recorded at approximately monthly intervals (mean ± SD = 27 ± 8.8 days; Supplementary Table S2) to quantify temporal dynamics of social relationships within the group. During each observation birds were recorded simultaneously with two HD cameras (GoPro Hero 3, GoPro Inc.) from different angles covering the vast majority of the aviary space. Videos (mean ± SD = 192.8 ± 49.1 min) were taken in the morning or afternoon after food was distributed (between 08:00 and 15:30 h, depending on the logistics of the field station). Later, one single observer (BH) scored the videos and recorded all dyadic social interactions using the software *BORIS* v. 2.995^48^. For each dyad, we recorded the identities of both the initiator and the recipient of action.

In a second step, we categorized behaviours into affiliative or antagonistic interactions following^20,49^. Affiliative behaviours included allopreening, collaborative foraging and exploring, seeking proximity or sitting close together. Agonistic behaviours included aggressive behaviours (e.g. pecking, feather pulling or jabbing), physical attacks, chases, and displacements of individuals (i.e. one individual approaches, resulting in another individual’s retreat from its current position; for details see Supplementary Table S3). Whenever an individual involved in an aggressive interaction was displaced or retreated it was classified as subordinate for this interaction. The interaction was classified as a draw, if no clear subordinate individual could be defined. We did not consider vocalizations for the categorization of behaviours and excluded ambiguous behaviours in which no clear affiliative or antagonistic intention could be observed.

### Vocal communications

Vocal communications between individuals involved in the attacks as well as other calls were extracted from the recorded videos using VLC media player version 2.2.4. Afterwards, we converted acoustic recordings into wave format and plotted time waves with the graphical function *oscillo* within the *seawave* package^50^ for *R* version 3.4.4^51^.

### Dominance hierarchies

For each day of observation, we separately obtained dominance hierarchies based on aggressive interactions using the Elo-rating procedure^52,53^. Elo-rating updates the ranks of individuals after each pairwise interaction by adding points to the winner and by subtracting points from the loser. This allows observing the development of dominance hierarchies over time. Initially, we set the score (so called elo-score) for each individual to 1000 and the number of points gained or lost each time to k = 100. Since some individuals interact more frequently than others, and because elo-scores are dependent on the temporal sequence of interactions, we repeatedly randomized the order of interactions and computed elo-scores 1000 times^54^. We then extracted the mean elo-score for each female, with more dominant individuals having higher elo-scores. All randomized elo-scores were computed by applying the *elo_scores* function in the *aniDom* package^55^ and hierarchies were visualized using the *ggplot2* package^56^ in *R*.

In addition to Elo-ratings, we constructed dominance hierarchies on the basis of David’s scores^57,58^, an index, which is independent of the sequence of interactions (for details see Supplementary methods). David’s scores were highly correlated with dominance ranks obtained from Elo-rating (Pearson correlation: r = 0.97, *p* < 0.001; Supplementary Table S4). We therefore only present dominance hierarchies obtained from randomized Elo-rating.

### Network construction

For each independent day of observations, we converted the recorded social interactions into two adjacency matrices, one with agonistic and one with affiliative interactions. In these matrices each row and column represented an individual and each cell specified the number of interactions an individual had with another individual. To account for differences in the total number of interactions caused by variation in the length of behavioural recordings (see above), we divided the number of interactions by observation time (interactions per hour). We then constructed two attribute-ordered networks^59^ for each observation day, in which nodes represent individuals and edges agonistic or affiliative interactions, respectively. All networks were weighted by the total number of interactions (width of edges) and directed. Networks were created using the *igraph* package^60^.

### Consistency of networks through time

To test whether individuals consistently interacted with the same partners among observation days, we applied a quadratic assignment procedure QAP,^61,62^. The QAP, which is comparable to a Mantel test, repeatedly permutes rows and columns of the dependent network matrices while keeping the observed relationship structure (i.e. strength of connections) between individuals^61–63^. After each permutation, the procedure recalculates the correlation, generating a distribution of coefficients. This matrix-specific distribution can then be compared to the observed correlation to determine significance^61,64,65^. We conducted QAP correlation tests separately for networks with affiliative and aggressive interactions and only compared social networks that included the same individuals. All QAPs were run with 1000 permutations using the *qaptest* function from the *sna* package^66^ in *R*.

### Ethical approval

All applicable international, national, and/or institutional guidelines for the care and use of animals were followed. Permission for sampling of wild carrion crows in Germany was granted by *Regierungspräsidium Freiburg* (Aktenzeichen 55- 8852.15/05). Import into Sweden was registered with *Veterinäramt Konstanz* (Bescheinigungsnummer INTRA.DE.2014.0047502) and *Jordbruksverket* (Diarienummer 6.6.18-3037/14). The housing of the study group and experimentation was authorized and inspected on site by *Jordbruksverket* (Diarienummer 5.2.18-3065/13, Diarienummer 27–14) and ethically approved by the European Research Council (ERCStG-336536). The current study was solely based on behavioural observations and non-invasive. Crows were hand-raised and thereafter tightly monitored by experienced staff including one animal caretaker present throughout the entire period of over 1.5 years. The social behaviour of the group was monitored on several occasions daily, from close-by during feeding and from outside the aviary. Health status was monitored on regular intervals by staff and external veterinaries. While aggressive behaviours over food access (e.g. threatening) or perching sites (e.g. displacement) could be observed, none of the crows showed aberrant behaviour or a visible indication of stress. Nor were any physical injuries observed until after the death of C59. Based on the videos, which were examined after the death of the two victims, the most dominant individual was kept separately from the other individuals.

## Supplementary information

Supplementary Information

Supplementary Information

Supplementary Information

Supplementary Information

Supplementary Information

Supplementary Information

Supplementary Information

## Data Availability

The dataset used for the current study is included as additional spread sheet as part of the Supplementary material. All videos from which the observation were extracted have been uploaded to figshare data repository and can be found under the following links: Video 1 (https://figshare.com/s/0e67fba459068b4c4916), Video 2 (https://figshare.com/s/c7bb61b67080fee12a0b), Video 3 (https://figshare.com/s/2f096f240cd99f977e77), Video 4 (https://figshare.com/s/7eaf0324cb1c91d6a3c1).
