# Author Correction: Dominance relationships and coalitionary aggression against conspecifics in female carrion crows

**DOI:** 10.1038/s41598-022-09406-3

**Published:** 2022-04-01

**Authors:** Benedikt Holtmann, Julia Buskas, Matthew Steele, Kristaps Sokolovskis, Jochen B. W. Wolf

**Affiliations:** 1 Division of Evolutionary Biology, Department of Biology, LMU Munich, Großhaderner Straße 2, 82152 Planegg-Martinsried, Germany; 2 Behavioural Ecology Group, Department of Biology, LMU Munich, Großhaderner Straße 2, 82152 Planegg-Martinsried, Germany; 3 Department of Ecology and Genetics, Evolutionary Biology Centre, Uppsala University, Norbyvägen 14-18, 75236 Uppsala, Sweden

Correction to: *Scientific Reports* 10.1038/s41598-019-52177-7, published online 4 November 2019

The original version of this Article contained an error in the spelling of the author Kristaps Sokolovskis which was incorrectly given as Kristaps Solokovskis. The original Article and accompanying Supplementary Information file have been corrected.

