# Supplementary material for: Dominance relationships and coalitionary aggression against conspecifics in female carrion crows
Source: Sci Rep. 2019 Nov 4;9:15922. doi: 10.1038/s41598-019-52177-7 (PMC6828704; doi:10.1038/s41598-019-52177-7)
Supplement: Supplementary file 3 — Supplementary Information [file 41598_2019_52177_MOESM3_ESM.pdf]

Supplementary materials
for

Dominance relationships and coalitionary aggression against conspecifics in female carrion crows

Benedikt Holtmann [1,2*], Julia Buskas [3], Matthew Steele [3], Kristaps Sokolovskis [3], Jochen B. W. Wolf [1,3]

[1] Division of Evolutionary Biology, Department of Biology, LMU Munich, Großhaderner Straße 2, 82152 Planegg-Martinsried, Germany

[2] Behavioural Ecology Group, Department of Biology, LMU Munich, Großhaderner Straße 2, 82152 Planegg-Martinsried, Germany

[3] Department of Ecology and Genetics, Evolutionary Biology Centre, Uppsala University, Norbyvägen 14-18, 75236 Uppsala, Sweden

***Author for correspondence:**
Benedikt Holtmann, Email: holtmann@biologie.uni-muenchen.de

Table of contents

Supplementary methods 2
Supplementary tables 3
Supplementary figures 8
References 10

Supplementary methods

Dominance hierarchies using David's score

In addition to Elo-ratings, we constructed dominance hierarchies on the basis of David's scores [1,2]. This alternative dominance measure is based on interactions matrices containing the total numbers of wins and losses of certain observation periods. Therefore, David's scores are independent of the sequence of interactions. We used the *DS* function within the *EloRating* package [3] in *R* [4] to calculate David's scores. To account for differences in the number of interactions between individuals resulting from individuals preferring or avoiding contact with certain conspecifics, we corrected David's scores for the number of interactions in a dyad [5]. David's scores were highly correlated with dominance ranks obtained from Elo-rating (Pearson correlation: r= 0.97, $p < 0.001$; **Table S4,** Additional file 1). We therefore only present dominance hierarchies obtained from randomized Elo-rating.

Supplementary tables

Table S1 Overview of carrion crow females that were housed together and video recorded between July and October 2015.

ID	ColourID	Colour bands	Species	Sex	Comments
C27	Grey	Black-Black	*Corvus corone*	Female	C27 re-joined the group on the 22nd of September after having been isolated for a period for reasons of health
C29	Blue	White-White	*Corvus corone*	Female	-
C45	Red	Blue-Blue	*Corvus corone*	Female	-
C58	Green	Blue-Green	*Corvus corone*	Female	-
C59	Yellow	Orange-Orange	*Corvus corone*	Female	-

Table S2 Days of sampling and length of video recordings

**Date**	**Time video started (hh:mm)**	**Length of video (hh:mm)**	**Crows present**
15/07/2015	08:00	04:30	C29, C45, C58, C59
18/08/2015	08:15	02:20	C29, C45, C58, C59
22/09/2015	11:45	02:55	C27, C29, C45, C58, C59
10/10/2015	12:10	03:26	C27, C29, C45, C58, C59
31/10/2015	11:25	02:53	C27, C29, C45, C58, C59

Table S3 Ethogram of affiliative and aggressive behaviours displayed by the observed female carrion crows. Behaviours were categorized following [6,7].

Type of interaction	Behaviour	Description
**Affiliative**	Allopreening (directed)	One individual preens or touches the feathers of another with its beak
	Seeking proximity (directed)	One bird approaches another individual with the intention to sit in physical contact (within the reach of the bill) to its "partner" or to check what the other bird is doing
	Dual exploration (mutual)	Two birds explore the same area and/or food items
	Dual foraging (mutual)	Two birds feed in close vicinity (sometimes from the same food item)
	Displaying (mutual)	Two individuals engage in a series of calling, bowing and tail-fanning<br><br>For more details, please see the subsection *'Recruitment'* within the section 'Coalitionary attacks and vocal communication' of the results in the main text
**Aggressive**	Aggression	One bird pecks, feather-pulls, or jabs after another individual
	Displacement (direct and indirect)	One individual approaches and causes another individual to retreat or to move from its position.

Type of interaction	Behaviour	Description
		Sometimes these interactions involved threatening gestures as well as vocalizations, such as “bristle-head posture”, (von Blotzheim Handbuch der Vögel Mitteleuropas), or aggressive calls
	Chase	One individual chases and tries to catch another bird.
	Attack	One bird attacks another in the air or on the ground

Table S4 Randomized elo-scores and David's scores estimated from pairwise interactions for each female carrion crows and observation day. The higher the score, the higher is the dominance of the bird within the group.

Date	Individual	Randomized elo-scores	David's scores
15/07/15	C45	1340.2	5.524
15/07/15	C59	1043.3	0.861
15/07/15	C29	934.7	-0.990
15/07/15	C58	658.4	-5.394
18/08/15	C45	1156.6	4.241
18/08/15	C29	988.4	0.687
18/08/15	C59	1040.2	0.247
18/08/15	C58	713.5	-5.175
22/09/15	C45	1362	8.565
22/09/15	C59	1172.8	4.250
22/09/15	C29	877.3	-4.126
22/09/15	C27	787.2	-4.479
22/09/15	C58	801.6	-4.210
10/10/15	C45	1271.9	7.544
10/10/15	C59	1097.6	4.119
10/10/15	C58	929	-3.063
10/10/15	C29	859.6	-4.580
10/10/15	C27	742.8	-4.020
31/10/15	C45	1508	9.461
31/10/15	C59	1197.8	4.716
31/10/15	C29	801.4	-1.012
31/10/15	C58	724.7	-5.023
31/10/15	C27	678.1	-8.141
15/07/15	C45	1340.2	5.524
15/07/15	C59	1043.3	0.861

Supplementary figures

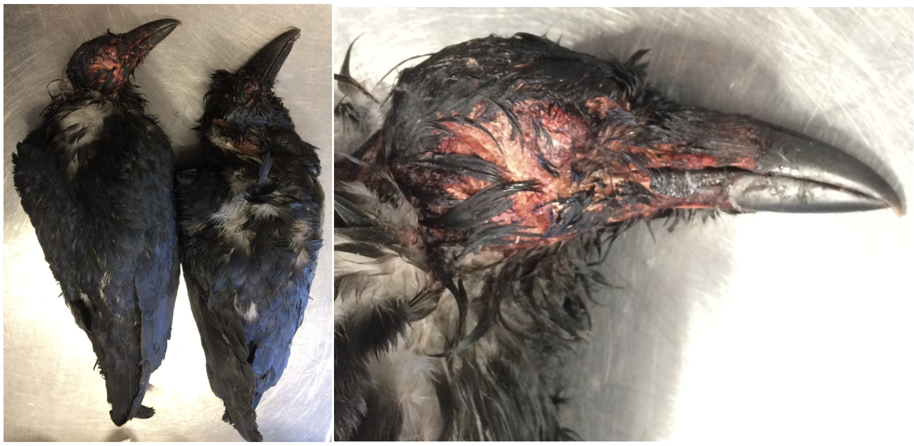

Fig. S1 The two carrion females (C27 and C58) that were found dead at the 1st of November and 3rd of November 2015, respectively. The left picture shows the two corpses of individual C58 (left) and C27 (right) that where sent to the National Veterinary Institute of Sweden for necropsy. Necropsies concluded that the two individuals very likely died due to traumatic injuries. The right picture shows a close up of C58's head injuries likely resulting from the coalitionary attack of individuals C45 and C59.

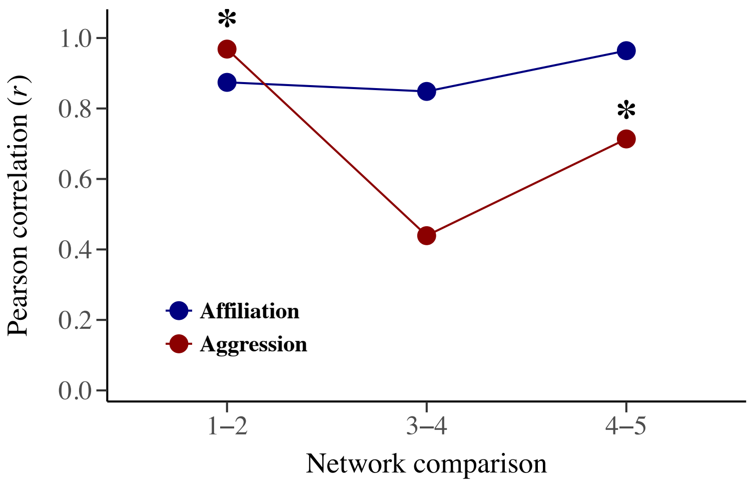

Fig. S2 Stability of affiliative (blue) and aggressive (red) networks over time. Correlations between two sequential networks (observation days) were obtained by comparing network matrix structure using quantitative assignment procedure (QAP) correlation tests. Network structures for aggressive interactions were significantly correlated for recordings 1-2 and 4-5 ($P < 0.05$). None of the affiliate matrix correlations were statistically significant ($P > 0.05$). The figure was created using the *R* package *ggplot2* [8].

References

1 David, H. A. Ranking from unbalanced paired-comparison data. *Biometrika* **74**, 432-436, doi:https://doi.org/10.1093/biomet/74.2.432 (1987).

2 Gammell, M. P., de Vries, H., Jennings, D. J., Carlin, C. o. M. & Hayden, T. J. David's score: a more appropriate dominance ranking method than Clutton-Brock et al.'s index. *Anim Behav* **66**, 601-605, doi:https://doi.org/10.1006/anbe.2003.2226 (2003).

3 Neumann, C. & Kulik, L. EloRating: animal dominance hierarchies by elo rating. *R package version 0.43*, https://CRAN.R-project.org/package=EloRating (2014).

4 R: A language and environment for statistical computing (R Foundation for Statistical Computing, Vienna, Austria, 2018).

5 de Vries, H., Stevens, J. M. G. & Vervaecke, H. Measuring and testing the steepness of dominance hierarchies. *Anim Behav* **71**, 585-592, doi:https://doi.org/10.1016/j.anbehav.2005.05.015 (2006).

6 Seed, A. M., Clayton, N. S. & Emery, N. J. Postconflict third-party affiliation in rooks, *Corvus frugilegus*. *Curr Biol* **17**, 152-158, doi:https://doi.org/10.1016/j.cub.2006.11.025 (2007).

7 Kondo, N. & Hiraiwa-Hasegawa, M. The influence of social dominance on calling rate in the large-billed crow (*Corvus macrorhynchos*). *J Ornithol* **156**, 775-782, doi:https://doi.org/10.1007/s10336-015-1191-8 (2015).

8 Wickham, H. *ggplot2: Elegant graphics for data analysis*. (Springer, 2016).
